# Principles of a New and Scientifically-Based Cranioscopy

**Published:** 1842-07

**Authors:** 


### Art. V.

*Grundzüge einer neuen und wissenschaftlich begründeten Cranioscopie.* Von Carl Gustav Carus.—*Stuttgart*, 1841. 8vo, pp. 87.

*Principles of a New and Scientifically-based Cranioscopy.* By C. G. Carus.—*Stuttgart,* 1841.

A period of little more than two years has elapsed since we felt ourselves called upon to enter at some length into an examination of the merits of phrenology, and of its claims to the respectful and attentive consideration of the members of the medical profession. In this examination we first took a cursory view of the actual estimate in which the subject seemed to be held throughout a considerable portion of the civilized world; we next discussed the character of its leading principles and some of the more interesting details, always attempting to keep in view the true value of the evidence upon which these rested; and in conclusion we expressed our conviction that, as a matter susceptible of practical application, it would be found, so far as proved to be true, of considerable importance and utility, not only in medicine, but also in many other things bearing upon the welfare of humanity. In this our advocacy, so to speak, of the real deserts of phrenology we would have it distinctly understood that our assent and our convictions extended only to that aggregate of natural facts which, in our belief, had been ascertained from correct and unbiassed observation, and only to those principles which constituted the expression of the general truths elicited by those facts. There are few sciences which have not suffered disfigurement and whose progress has not been seriously retarded by inaccurate observation and hasty generalization; and it was *à priori* to have been expected that phrenology, however true in its foundation, should, in the erection of the superstructure, be subjected to the same hinderances and causes of misapprehension, to the same admixture of inaccuracy and imperfection in the detail, and to the same confusion of mere hypothesis with true logical deduction, as more or less happens to almost every science, especially when in its infancy, and when struggling for general recognition. We conceive, indeed, that this has been the case with phrenology to a more than ordinary extent; and to this cause we mainly attribute the great backwardness shown in so many instances by scientific men to a fair and candid examination of its true merits. We propose in the present article, before referring to the work with whose title it is headed, to offer a few remarks upon the present state of phrenology *as a science*—as an accumulation of facts developing principles—upon some of the causes which, in our opinion, have retarded its progress *as a branch of physiology*—and upon the necessity of its being prosecuted more in the spirit of a true inductive philosophy than has hitherto been exhibited by many of its more enthusiastic and popular expounders, if it is to emerge from its present infantile condition, and to obtain the bold and defined outline of a well-matured science, commanding, not soliciting, the attention of those to whom its truths are of practical importance.

Notwithstanding the antiquity of the notion that the brain was, in some way or other, the constant associate of the intelligence; notwithstanding this notion received confirmation from the disquisitions of almost every philosopher of the ancient world, and illustration from the labours of a more practical and experimental age; we hesitate not to affirm that, as a proposition resting upon widely-extended observation, as a principle deduced from the universality of the fact, it is entirely within the domain of phrenology. Anterior to the teaching of Gall, it was most customary to regard the brain merely as the source and centre of nervous influence, distributing the same, through the medium of the nerves, to the rest of the system. We here allude, not to the speculations of metaphysicians, but to the doctrine taught in the schools of anatomy and physiology. Witness the nature of the objections proposed by individuals of no mean repute to the discovery of Gall at its first promulgation. Did not these, in some cases, professedly rest upon certain assumed facts tending to show that the phenomena of mind could be displayed, in all their integrity, in the event of partial or even complete disorganization of the cerebral structure? Is not such a line of argument, moreover, adopted by many at the present day? The truth is, neither metaphysician nor physiologist attempted, in anticipation of Gall, even by hypothesis, to establish a precise and definitive relation between the mind and the brain, after the manner of the leading proposition in phrenology. Mind, in the language of phrenologists, includes within the designation not only the intellectual faculties, but all the affections and sentiments proper to the principle of consciousness—in other words, the conscious principle itself, with all its attributes; whereas, in the hypothesis that had been hazarded relative to the association of mind and brain, the former term was almost universally restricted to the intellectual faculties: but even had this been otherwise, the establishment of any truth by processes agreeable to a rigid philosophy of induction alone constitutes the merit of discovery. Distinction between the functions of sensation and voluntary motion had long suggested the idea of separateness in the subservient nerves, and pathological facts had strengthened the idea; but confirmation by demonstration, and the establishment of the great principle which it tends to develope, belong most assuredly to modern physiology. In a similar view of the case, we must reiterate our decided conviction that the proposition, that the brain is the organ of the mind, forms peculiarly a principle of phrenological science, and that, however general the assent yielded to the same in the present day, even by parties who would disdain to be considered phrenologists, it is not the less a principle strictly phrenological. Gall was the first who interrogated nature, in all her departments, to ascertain the fallacy or soundness of the principle in question; and he was the first successfully to investigate certain facts that had seemed to militate against the proposition, and to show their entire accordance with the general rule. Hence we conceive that whoever admits the function of the brain to be to develope the attributes of the conscious principle is, *pro tanto*, a phrenologist, and a disciple of Gall. In fine, phrenology, as a science, has, in our estimation, established, by the method of induction, the soundness of its first principle, that *the brain is the organ of the mind.*

The next leading proposition in phrenology, which sets forth that separate parts of the brain are instrumental in manifesting the individual faculties of the mind, we hold also to be quite as well supported by the evidence of facts as the great majority of recognized truths in nature. We believe that the extensive observations made by Gall and his disciples, not only upon man, but upon the animal kingdom at large, have clearly made out that, in proportion to the complex and multiform character of the cerebral hemispheres, the species or the individual ranks high in respect of psychical endowment; that in those species where the presence of any structure really analogous to the cerebral hemispheres is wanting, there is no clear or certain manifestation of the principle of consciousness; that in fishes, where evidence of this to a slight extent is noticed, an extremely simple development of brain is discovered; that in birds, where an obvious addition of mental power takes place, a correspondingly more complex character of the cerebral hemispheres is coincident; and, lastly, that in pursuing the anatomy of the brain through the whole class of mammalia up to man himself, a continuous illustration of the same fact is to be found, suggesting the inference, in our estimation most philosophically, that multiform character and complexity of these masses are related, in the way of cause and effect, to variety and extent in the faculties of the mind; and, consequently, the second principle in phrenology becomes fixed, that *the brain forms a congeries of organs, the function of each being to manifest some faculty of the mind.*

The third principle insisted upon by the phrenologists, that which embodies the whole soul of the new doctrine, and that without the satisfactory establishment of which the whole system would lapse into chaos, like an arch on the withdrawal of its key-stone, flows from the universality of the fact, that *size of cerebral apparatus*, cæteris paribus, *constitutes a measure of functional power.* It was the observation of facts illustrating this principle that laid the foundation of phrenological science, and we believe that in the progress of such observation many general truths have been ascertained. We are convinced that any one who will take the trouble to look at what occurs in nature will be satisfied that, under certain conditions detailed at some length in our former article, an ample, well-developed forehead, indicating great size of the anterior convolutions of brain, is the constant associate of high intellectual power; that a broad and elevated sincipital region ever characterizes the heads of individuals eminent for the excellence of their natural dispositions; and that a large mass of brain immediately above and behind the ears shows the possession in a great degree of the qualities communicating to the individual animal energy. Notwithstanding the difficulty of obtaining clear evidence or even illustration of these truths from every case, we are quite certain that if extreme instances be taken—and this is always necessary when the mind has to be convinced of something new—we are quite certain, we say, that the facts in question will be observed to be universally true, and, for this reason, calculated to settle, upon a sure basis, the third principle of phrenology. The same thing will ever obtain, both in comparison of individuals and species, provided certain qualifying circumstances are kept in view, which our present limits will not allow us to discuss, but which are qualifications that enter into the estimate of physiological facts in general, and therefore not rightly to be charged upon phrenology as so many loopholes of escape—a proceeding sometimes indulged in by parties directing only a superficial attention to the subject.

In forming an estimate of the present condition of phrenological science, as a system expressive of certain general truths deduced from the consideration of natural phenomena, it might not be out of place here to enter into an examination of the more minute details; to determine, so far as practicable, the particular cerebral organs (to use the ordinary phraseology) which, from the extent and character of the observations, may be regarded as *established;* to specify those the proof of which is yet defective, and which consequently are but to be deemed *probable;* and, lastly, to discuss those which are merely *conjectural.* This, however, would carry us much beyond our allotted space; and we must content ourselves therefore with stating in a few words that, after a fair amount of attention bestowed upon the subject, we are satisfied that, in the case of many of the cerebral organs, it will be found that a conspicuous development of the same will *always*, under given circumstances, be found associated with great vigour in the corresponding faculty or disposition; that in the instance of some others ordinarily assumed in popular works upon phrenology, we believe them to rest upon observations which are either too imperfect in their character or too limited in their amount, yet to rank amongst the truths of science; and lastly, that a great deal which is readily and eagerly embraced by the more enthusiastic and the less thinking of professed phrenologists, deserves, in our opinion, little more consideration than that which is due to so much pure speculation.

Over-zealous and injudicious friends are ever more effective obstacles to the advancement of any new science than its professed opponents. We believe that in nine cases out of ten where truth is rejected, it occurs from misapprehension of the real character of that truth; and when ignorant zealots, in any cause which they afflict with their advocacy, misstate facts and overstrain inferences, they furnish enemies with most powerful weapons of attack and at the same time disgust the impartial and the sober-minded, deterring them from that investigation to which a simple exposition of the real merits of the case might have led them. It has happened so with phrenology. A great proportion of its popular expounders, in lectures and in publications, have presented it to the public as a science that had unravelled every mystery of man's moral nature, and as something that had simplified to the utmost possible extent the nature of the mind's dependence upon organization—as a key to unlock the secret recesses of every heart—and as a system which, whilst it might enable the bad to render it subservient to selfish and evil purposes, would yet furnish the philanthropist with means for the regeneration of society far transcending any that had heretofore been at command. We do not think that we exaggerate in this account of the ridiculous pretensions of many of the *soi-disant* phrenologists. We do not of course refer to the works of the more celebrated and philosophical writers on phrenology, though we have sometimes wished that the labours of the leading cultivators of this science had rested more exclusively in the field of physiology and natural history, thereby following in the track so successfully beaten by Gall, whose exertions we very much think with Dr. Elliotson, brought the new physiology of the brain nearly to its present mark, *as a science of facts.* We should have been glad to have seen it a little more matured and extended in many of its details ere so many of its truly meritorious advocates had drawn so largely upon it for practical application. To illustrate this, however, is not exactly our present business. It is notorious that many writers and lecturers upon phrenology have taken up the subject, not from any patient and detailed examination of its evidences, but from their fancying it reconcilable with, and confirmatory of, anterior prepossessions, or from the facility it has seemed to afford of exploring hitherto inaccessible regions. This would be a matter concerning themselves only, if it did not happen that the progress of science becomes thereby arrested; adversaries, from ignorance or less creditable reasons, directing their attacks to the subject as propounded by such persons. We have heard and have read much in opposition to phrenology, and yet we can affirm that the phrenology opposed was scarcely ever that of Gall, but usually its miserable caricature exhibited by half-informed enthusiasts.

Popular phrenology has indeed exceeded all reasonable limits of a sound philosophy. When we remember that, in its legitimate pretensions to rank among the sciences, it is essentially a department of physiology—that which explains the functions of the brain—we must greatly regret that its exposition and pursuit have fallen so much into the hands of persons whose accessory knowledge has but little qualified them for the prosecution either of science in general, or physiology in particular. In this state of things, which for the sake of truth cannot be too much lamented, one can hardly feel surprised that this branch of science has been cultivated by many much more in the spirit of speculative advocacy of a favorite set of opinions, than in that of an honest desire to obtain a correct acquaintance with the economy of nature. What do we find upon opening three fourths of the treatises extant upon phrenology? Assuredly, to any one whose mind was new to the question, it would seem, from their perusal, as though phrenology were the most perfect of the sciences, rather than as one professedly in its infancy. We shall see, probably, in such treatises, the brain systematically divided into its thirty-six organs, the functions of which shall be stated with as much confidence, and with the same terseness and abruptness of language, as if there were question of the five external senses; scarcely any distinction being drawn between the established, the probable, and the conjectural; the primitive destination or final cause assigned to each, not in the way of conclusion arrived at from a consideration of all the allied circumstances, but as part of the general demonstration; then we have the doctrine of the temperaments paraded, as if *that* were peculiarly a branch of phrenology, and as if there were some positive knowledge upon the subject—which there is not; and altogether there is in the affair of fact so much error and over-statement, in the detail such confusion of demonstration and hypothesis, that that which should constitute the highest branch of physiology becomes really so degraded as to seem to merit exclusion entirely from the domain of science.

If in the representation of the actual facts of phrenology there is so much to reprehend, assuredly the matter stands worse still in the attempt at obtaining conclusions. Any suggestion or conjectural statement at any time hazarded by some leading phrenologist is, to serve the immediate purpose, referred to and employed as so much positive demonstration. Whatever be the favorite bias of thought of the advocate is sure to receive its strongest proof from the science at present under discussion,

Materialism and spiritualism, fatalism and moral liberty, revealed religion and pure theism are all in turns established incontrovertibly by popular phrenology. Thus, the philosophical phrenologist, in discussing the nature of man, aided by the new physiology of the brain, shall occasionally give emphasis to his words by employing the phraseology of the sacred volume, when immediately will arise some of your petty phrenologists, and contend that religion and its ministers may henceforth be dispensed with, because phrenology teaches the same things as the really excellent parts of scripture, and much more clearly; as if the reasonings of phrenologists, aided by the help of revelation, were something inherent in phrenology, and something deduced solely therefrom. The same process obtains with respect to medicine, politics, legislation, and education. Because useful hints and, in some instances, even new information may be gathered from the subject bearing upon some of these matters, therefore all that is advanced or enforced by the collateral aid of phrenology is phrenology itself! Any one who has paid much attention to the sayings and the doings of the vulgar herd of those whom Gall would have disdained to own as his disciples, will recognize a faithful representation in what we have here advanced. As expressed in our former article upon this subject, we are confident that, in the prevention, discrimination, and treatment of insanity and nervous diseases, in education and also in legislation, in the prevention of crime and the treatment of criminals, phrenology will, when freed from many popular errors, furnish very useful aids and suggestions; but to expect that a perfect revolution in dealing with all these things is, from such source, to be at once accomplished, is verily but the day-dream of the enthusiast, or the shallow pretence of the charlatan. We, for our own part, coincide very much with the sentiments perspicuously expressed by Dr. Cowan, of Reading, in a paper published in a recent volume of the Transactions of the Provincial Medical and Surgical Association, viz. that phrenology, as such, "does not involve that addition to our *real* knowledge of the nature and laws of mental and moral phenomena which some able writers seem to imply, since it must be admitted that much general knowledge had been previously acquired," and which general knowledge phrenology does not by any means supersede.

In reviewing the circumstances which have tended to lower phrenology in the estimation of scientific men, and consequently to retard both its progress as a science, and the general recognition of its leading truths, we should but very imperfectly perform our task if we did not refer, in the strongest possible terms of reproof and condemnation to the too-prevalent proceeding of examining living heads in minute detail and indiscriminately, and supplying the owners with an account of the "development," often on the receipt of a fee, varying in amount as there is furnished or omitted a general deduction as to the character and probable conduct of the individual, with or without the "philosophy," according to the phraseology of practitioners of this art. The attempted justification of this course rests upon the plea, that evidence of the truth of phrenology becomes thereby afforded, and that the most useful of knowledge—self-knowledge—is, at the same time, gained to the individual. What, however, is the real state of the case? Why, we unhesitatingly maintain, that the science is not sufficiently advanced to supply evidence of its truth from every head, or from any one head; and consequently that such practice, as a general one, is so much pure charlatanism. Where any strongly marked peculiarity of individual character exists, its outward sign, in appropriate subjects, will certainly be detected; but, from the very nature of the thing, these cases must constitute, not the rule, but the exception. The practice we condemn, however, makes no distinction of instances. Injudicious zeal—the common ally of ignorance, a wish for effect, not unfrequently more sordid motives, stimulate the self-styled phrenologist in this empirical career; and, as a matter of course, the errors and mistakes perpetually made are constantly appealed to as indicative of the sandy foundations of the entire phrenological edifice. We write advisedly in this our unqualified reprobation of the popular custom of "taking developments." We believe it to be an extension of the practical application of phrenology much beyond its legitimate bounds; and we appeal to any one having acquaintance with its results, whether anything like uniformity—the true test of accuracy—is obtained in the majority of cases, even when the most experienced and dexterous pronounce their judgment, if their explorations be conducted separately. We ourselves have even witnessed the greatest possible discrepancies. Nay, we have seen the *same* phrenologist furnish one character from the head and a totally different one from the cast, whilst in ignorance of the original of this latter. This we have known to happen, not merely in the practice of one of your shilling-a-head itinerants, but in that of one not unknown to fame in the annals of the science. It may be contended, that mistakes on the part of the manipulator no more indicate the inaptitude of phrenology for the objects with which "taking developments" is practised, than do those of the chemist or the astronomer show the unfitness of their respective sciences for exhibiting certain results. This objection at first sight seems plausible, but a closer examination will show its futility. It must be borne in mind that here is question, not of erroneous or premature inferences to which an experimental research into natural phenomena may sometimes conduct even the most philosophical enquirer, but of the legitimate and authorized application of a science as it stands; and in this view of the matter we may assert, without any fear of contradiction, that mistakes occur, as the exceptions, in the progress of *illustrative* experiment in such sciences as chemistry and astronomy, at least in the hands of persons eminent in their respective departments, and are, moreover, easily rectified so as to prevent their recurrence; but in the case of *indiscriminate manipulation of the living head for the purpose of exhibiting evidence of the truth of phrenology*, blunders, and gross ones too, are perpetually arising, and that in the practice of every one, no matter how experienced. We do not speak rashly in our present assertion. We have seen many certificates of character from phrenological artists, with which the parties for whom they were drawn out have been quite delighted, and which have constituted the proximate cause of their conversion to the true phrenological faith, but which have ever seemed to us merely as a sagacious dwelling upon two or three actually possessed and really indicated peculiarities, mixed with a vast amount of generality that would apply to almost any case, and the whole dished up with a seasonable amount of flattery; which latter ingredient has commonly furnished the relish for the whole affair, ultra phrenology into the bargain. It is degrading to an important—we believe the most important department—of physiology to solicit or obtain *such* converts by *such* means. Non tali auxilio, &c.

We regard, then, the unscientific character of a large proportion of those who have devoted themselves to the study and the exposition of phrenology, the over-statements and the mis-statements as to its ascertained facts, the extravagant inferences therefrom, and the confounding of these latter with the positive demonstrations of the science, and, above all, the empirical procedure in the matter of taking developments, as constituting the principal causes that have interfered with its cultivation as a branch of physiology, and retarded its progress to recognition on the part of those whose suffrages it is always desirable to obtain in that stage of a new science wherein its conclusions seem most to oppose themselves to anterior knowledge or prepossessions. To a certain extent, we believe that all this was inevitable from the very nature of the subject. Phrenology, at first view, addresses itself too much to that spirit of wondrous inquisitiveness prevalent in little minds not to have attracted the attention, and excited the interest, of the sciolist and the vain-glorious; and, as a natural consequence, we have had the misappreciation and the misapplication of the matter, such as we have just detailed. Let, however, the really profound, the *true* phrenologists, shake off the shallow and contemptible allies with whom they have been too long encumbered, and too much identified; let them disclaim their superficial unscientific modes of investigation; and let them pursue their own continued researches as much as possible in the spirit that actuated Gall.

Before offering any suggestions as to the way in which we believe it to be desirable to prosecute future investigations into the physiology of the brain, we would just point out in what way it has happened, in our opinion, that, except in diffusion and illustration, so little has been done, since the period at which the great discoverer closed his active and successful career. We attribute this circumstance in some respects to the premature introduction of speculative reasoning, by the earlier disciples of Gall, into the exposition of that which had been really ascertained. *Deduction* from principles already obtained, prematurely interfered with the *induction* of new truths; the former process being, at all times, the more captivating to great numbers, from its comparative facility. Thus, notwithstanding the credit that really attaches to the late Dr. Spurzheim for his share in the prosecution and exposition of the new physiology of the brain, it must be granted that his labours, after separating from Gall, rested but little in the field of active observation, except for the purposes of confirmation and illustration. We are aware that several important additions to the positive facts of phrenology are usually considered to have been made by Spurzheim. These, as a whole, we have long thought have too readily been admitted. We cannot go into detail, but, with the exception of one or two instances, we, for our part, regard these "additions" only as falling within the limits of the probable. Our present object, however, is to refer to the *method* pursued by Spurzheim, which we consider to have operated more or less disadvantageously upon the labours of his successors. From all that we have been able to gather from the published accounts of the assumed discoveries, made by the distinguished colleague of Gall, it has always appeared to us that nature was appealed to, or interrogated, not so much with the design of educing a general truth from the aggregate of the facts observed, as for the purpose of gaining support for a preconceived hypothesis. If we take the organ of "conscientiousness," a sentiment which Gall identified with that of "benevolence," *reasoning* led Spurzheim to dissociate the former from the latter feeling, and to infer the existence of a separate division of the brain as its material instrument, and an appeal to a very few *recorded* facts was made to obtain a realization of the idea. Our own conviction is that, in this case, the *anticipation* of nature, to use the phraseology of Bacon, suggested the right *interpretation;* and we quarrel not with the result, nor yet with the method, in the individual instance, but we refer to it as having operated prejudicially in the way of example; for, of late years, whenever there has been an assumption of discovery in phrenology we have generally noticed that the first suggestion had originated much more in speculative reasoning than in observation, and that cases in corroboration were most commonly few in number, and *selected;* whereas, in the proceeding of Gall, whom, in the language of the late Mr. Chenevix, Bacon might have rocked in the cradle, facts in large amount, *indiscriminately obtained,* were constantly amassed ere he would entertain a suggestion; and only after observations had accumulated extensively, would he venture to pronounce that any proposition was made out. He himself informs us that, on some occasions, after his collected facts had for years pointed to a particular conclusion, the occurrence of one certain unequivocal exception ever induced him to start anew. It is in this spirit, in that of a true inductive philosophy, that we would see future researches into the functions of the brain pursued. We think that physiologists and natural historians would do well to leave for a while the examination of the fundamental powers and special destination of the particular faculties, and somewhat more actively and generally resume the comparison of the *actions* of man and animals with their respective organizations. After all, it is only the results obtained in this way that can ever command general assent: the expression of the universal fact can alone form an axiom not to be shaken. Whatever is done in the way of defining and explaining the primitive intention, or design, of the mental powers and affections, can impress the mind only, at best, as a highly probable conclusion, never as an absolute demonstration. These constitute very appropriate themes for the mental or moral philosopher who, in the prosecution of his studies, thinks it well to draw upon phrenology; our business at present with this latter is, however, chiefly as *a branch of physiology;* and it is to the subject regarded in this point of view that our present remarks are intended to apply.

We have often thought that that which has sometimes been attributed to Gall as his greatest mistake was, in point of fact, in the infancy of his investigations, a leading merit. We allude to his designation of many of the cerebral organs, according to actions that had been *motived* (if we may borrow from our Gallic neighbours) by irregular impulses, rather than according to the legitimate direction of the faculties. To have done this latter, in the early stage of his labours, would necessarily have partaken more or less of the spirit of the system, a plan irreconcilably at variance with the successful search after a new truth. Gall's course was to notice great developments of parts of the brain in combination with energy of function, as manifested by actions; and, constituted as human nature is, this was of course pursued best, in many cases, in the instances of great abuse; and, by adopting a nomenclature that simply expressed the general fact observed, he avoided that very common and almost inevitable source of error, premature explanation. The proceeding was perfectly philosophical, and had the advantage not only of keeping his disciples within the limits of demonstration, but of pointing out, at the same time, the only method by which the subject could be successfully followed out by those who should come after him.

The foregoing is the only method, we firmly believe, whereby phrenology, as the physiology of the brain, can be advanced or perfected. Extensive observations yet require to be made, in order to establish the precise set of actions, or mental manifestations, constantly associated with great development of several of the organs, usually represented upon the marked bust as though they were quite ascertained. Respecting these, we have as yet much disagreement amongst professed phrenologists, as to the nature of the primitive function they are intended to perform. Would it not be well if it were determined, first, what are the outward efforts, or displays, *invariably* associated with the given development? As an example, let us adduce the case of that which has been most commonly designated the organ of wit: suppose that some phrenologist should tax himself with the discovery of its exact function, we conceive that it would be well, instead of indulging in metaphysical refinement by the aid of a few *selected* facts, to collect records of the mental peculiarity of *every* individual encountered who was characterized by the development in question; and, when the accumulation had become considerable, to examine the records in order to see what was common to every fact in the series, and the result might go far to suggest the special manifestation of the organ in question. If this proceeding were followed up by obtaining *indiscriminately* negative instances in large number, and if it were found that in *all* cases of deficiency in organic size an absence was detected of that which had distinguished the positive instances, the proof we imagine would then be tolerably complete.

We are of opinion that, in phrenology, observations lose much of their value, as suggesting anything new, when they have been made by different individuals, one having taken the idea from another, and a few only having been contributed by each; because there are circumstances, in such observation, which render it very difficult to examine their value as *comparable* facts: whereas the case is different with an extensive amount collected by one *good* observer, as here the instances all possess the recommendation of having been estimated by the same mind, of having been measured, so to speak, with the same instruments; and, in this case, they afford a reasonable presumption of constituting properly comparable examples.

The proceeding which we have endeavoured to point out, and to illustrate, would simply form the counterpart of the labours of that one observer whose results were so signally successful; it would merely be an application of the method of induction. Whatever, as fact, is imperfectly proved in the present state of phrenology should be submitted first to the test of this method; and, on the attainment of any inference, facts in natural history, pathology, and practical medicine, might all be appealed to for confirmation; but, for the actual discovery, whatever should form the occasion of the first *suggestion*, assuredly the *proof* must ever be established in this way.

In cursorily reviewing the existing state of phrenological science, the causes of its too common neglect amongst scientific men, and the mode in which we believe its study should be hereafter prosecuted in order that it should be advanced and perfected as a branch of physiology, there are many circumstances and details into which our present limits will not allow us to enter; otherwise, there are some matters relating to all these points upon which we might have wished to dilate. We might have extended our remarks, somewhat, in referring more particularly to those parts of the brain whose function we considered to be pretty well made out; we might have *named* the organs, so called, in our belief established; those but probable and why; and those which we regarded as purely conjectural and the suggestions of a speculative spirit. We should have wished to notice more at length the custom of presenting a theory of the temperaments, as furnishing signs for the appreciation of the *quality* of cerebral structure, almost as accurately as prominence in the various regions of the head gives the clue to the detection of *size* of particular parts—a course pursued in the most confident and dogmatical manner by the vulgar herd of phrenologists. We should like to have spoken also upon the proposition, which some think they have demonstrated by experiment, that an alteration in the form of the head, caused by increased development of the particular organs, can be induced, even in adult age, by a *voluntary* action of the individual upon his own mental operations. These topics, however, we must omit, simply expressing our wish that, for the sake of what is really true in phrenology, enthusiasts and zealots would not press prematurely into the service what only rests upon data confessedly imperfect and, we believe, in great part erroneous.

Having long felt dissatisfied with much that we conceived to be unscientifically based in *popular* phrenology, we confess that it was with some feelings of interest and curiosity that we took up a work professing to teach the "principles of a *new* and scientifically-based cranioscopy"—the pretension with which the author, M. Carus, in his title-page, offers his system to the world. Before giving our readers any account of the contents of this work, we cannot refrain from expressing our surprise and regret that a man possessing a good reputation as a human and comparative anatomist, like M. Carus, should have identified his name with a series of propositions so thoroughly unscientifically based as those contained in the present production. We have here an additional fact, showing how wretchedly an individual may *reason*, who shall yet *know* a great many things; how an extensive acquaintance with details does not necessarily lead to right dealing with principles. M. Carus is, we believe, considered by some and, we suspect, especially by himself, as possessing a genius that is universal; and in his case we have an illustration of how incompatible, except in some rare instances, such an order of mind is with accuracy and precision of thought.

The new cranioscopy, inculcated in the little volume before us, rests essentially upon a threefold division of the contents of the cranial cavity —into the cerebral hemispheres, the corpora quadrigemina, and the cerebellum. These he speaks of as the anterior, the middle, and the posterior cerebral mass; to these three divisions he regards what he calls the three cranial vertebræ—the frontal, parietal, and occipital bones—as corresponding. Certain analogies in the lower tribes of animals have guided him in the formation of this view of the matter. Pursuing the subject, he places the intelligence in the cerebral hemispheres, the sensibility (Gemeingefühl) in the corpora quadrigemina, and the will, desire, and instinct of generation, in the cerebellum; and he tells us that herein "lies the key to all cranioscopy that is true and supported by physiological principles." His ideas upon the cranioscopy or phrenology which recognizes Gall as its discoverer appear to have been taken from some of the humblest and least informed of the modern phrenological school, or, what is worse, from the representations of some scoffing opponent, as he goes on to observe that "most other relations specified by Gall and his successors, and especially the presumed relation of individual moral qualities to certain bony prominences are thoroughly illogical, unphysiological, and untenable. It is just to these hypotheses, however, that the multitude have the most resolutely clung. It was hoped that, in such representations, a means was to be found for ascertaining whether any particular individual were good, benevolent, religious, or imaginative, whether he were quarrelsome, cruel or thievishly disposed, and so on; in the cases of children, that one might be able so to grope out their special talents and inward vocation as to regulate their education accordingly. All that sort of thing has its proper place among dreams and imaginative conceits." This extract from the early pages of the work under review will form a fair specimen of the style and spirit of the whole.

We shall not engage the time and the attention of our readers with anything like a detailed analysis of M. Carus's scientific cranioscopy. We shall content ourselves with pointing out some few of its gross inconsistencies, and its utter variance with all sound physiological demonstration or reasoning. We presume that when our author speaks of that which he calls "scientific" he does not propose to institute a new system of philosophy as well as of cranioscopy, but that he recognizes the validity of the Baconian system, as applicable to the investigation of the economy of nature. As then, in the progress of his work, he distinctly acknowledges the influence of organic size in the communication of functional energy, we should like to ask him whether it be a constant and universal fact that, according to the development of the cerebral hemispheres, the "intelligence" of the individual is exalted; that, to take his own nomenclature in specifying the faculties appertaining to the intelligence, Conception (Vorstellen), Perception (Erkennen), and Imagination (Einbildung), are in contemporaneous strength and activity, as the brain proper, being healthy, is large or small? A very few instances, taken indiscriminately, would disprove such a position at once. If we proceed to the next conclusion deduced by M. Carus, that feeling is associated with the corpora quadrigemina, where is the proof that energy or intensity of this characteristic bears any such relation? A few vague analogies, admitting of various interpretations, collected from the animal kingdom, assuredly constitute no "scientific" basis for any physiological proposition. Again, in regarding the cerebellum as the organ of the Will and Desire, (Wollen, Begehren,) as well as of the sexual instinct, where is the shadow of proof physical or metaphysical? Have men strong determination and resoluteness of purpose, in the general sense, ***always*** in relation to the development of the cerebellum? Where is the evidence that desire, the ***felt requirement*** of any individual, apart from what relates to sex, is associated specially with this structure, or coincident in any way with the strength of the will? We shall see, by and by, the proofs which our transcendental philosopher adduces. Meanwhile, let us ask, could a more unsatisfactory series of propositions have been brought forward? Could a more meager, inconclusive analysis of the psychological principle have been offered?

We proceed now to state the mode in which these notions are supported. We are told then that a large forehead, for the proper estimate of which minute directions are given, indicates high intelligence, and an appeal is made to examples for corroboration of the assertion. Here we are very much agreed with M. Carus; but we utterly deny that size of the frontal bone furnishes any measure of the magnitude of the cerebral hemispheres in the aggregate, which, he however maintains, constitute the organ purely of the intelligence. We believe that we should be wantonly consuming the time and the patience of our readers were we to commence a formal confutation of so absurd a conception. Next, after having been given to understand that Feeling, in what precise sense our author very imperfectly defines, resides in the corpora quadrigemina, and that these are developed according to the "dominance of the vegetative life, and of the individual feelings, without enlightenment by knowledge and without force of will;" we are directed to ascertain the magnitude of the region inclosed by the parietal bones in order to ascertain the preponderance of the qualities allocated in the "middle cerebral mass," as he calls the corpora quadrigemina. The middle region of the brain is certainly in excess over the anterior and posterior regions, when the fulness and superficial extent of the bones by which it is covered are proportionately great, but it is the middle lobes of the hemispheres, and not the twin bodies, that fashion this portion of the outer head. It would be pure supererogation to go into any defence of our assertion.—Power of will and strength of desire are to be inferred when a large occipital region is discovered, according to M. Carus, as if the magnitude of the bone inclosing this portion of the brain, furnished a rule for estimating the size of the cerebellum, where, according to our author, these qualities have their habitation.

The above paragraph furnishes the groundwork and outline of the new theory proposed by M. Carus; and, in the little volume before us, it is wrought out by a number of illustrations and extensions of its application, to go into which would be thoroughly useless. We have given the present slight sketch, neither from believing it capable of yielding any useful information nor from a desire to amuse the reader, but from the honest wish to warn the *orthodox* school of phrenologists, so to speak, from attempting premature conclusions and from building upon vague analogies; since it is apparent that such a method, once in vogue, may lead to the formation of another and a new phrenology, which, however, in the present instance, we regard as too transparently at variance with the plainest facts to have any chance of creating a school of *new* phrenologists. Still a more plausible scheme, one a little more consistent, might share a luckier fate; and would it not become an exhibition truly deplorable to behold rival systems vieing with each other in contention for disciples? And, indeed, if orthodox phrenology is to be disguised and disfigured by half-proved statements, empty conjecture, and foolish reasoning, we see not why a new phrenology should not constitute a formidable competitor. It would be, to a great extent, hypothesis and speculation in opposition to hypothesis and speculation, and fancied resemblances would determine the decision.

A disposition to argue from analogy, which too often leads to a neglect of the steady inductive mode of reasoning, we believe to be at the foundation of most of the errors that arise in science. Too great an inclination to deduce an inference from facts not standing in direct relation to each other can alone explain how it comes to pass that a man of M. Carus's extensive acquirements and experience, can have imagined for a moment that his own crude and most inconsistent ideas were either to supersede the philosophical results of Gall's labours, or, in opposition to them, to be regarded as a system "scientifically based." Further, we are sure, from the whole style of the work, that the author has no correct acquaintance with phrenology, but that his notions respecting the same are obtained, either from the representations of some inimical scoffer or from the readily-perused account rendered of the same by some half-informed and shallow zealot. In either case it is most unworthy of any man of science to attack the discoveries and to deride the philosophy of others, himself being in ignorance of what these discoveries truly consist, and of what this philosophy is; and more particularly when this is done in a work heralded by a high-sounding and loftily-pretending title, and which work shall not contain a single original observation, and the philosophy of which shall be made up entirely of speculation.

We must, however, take our leave of M. Carus; and, in doing so, we must express our conviction that the department of comparative anatomy is but little calculated to afford the means of *discovery* in phrenology. In the case of all physiological discoveries, function has not been revealed by dissection of parts, nor by careful study of their forms, dimensions, or their analogies. This has always been accomplished by noting the phenomena of life and the conditions under which they have been displayed; and then anatomical facts have been investigated in order to illustrate still further and render more clear the information obtained by the observation of the living action of organs. When anatomy is appealed to for the *proof* of any assumed novelty in physiology, it will ever fail, though it may occasionally supply the first hint. If this be true of anatomy and physiology in general, assuredly the matter is not altered when there is question of cerebral physiology and comparative anatomy. If notions are to be taken up resting only on certain real or supposed analogies existing amongst various tribes of the animal kingdom, and to be regarded as "scientifically based," we do not see why there may not be as many systems of physiology as there are different students, for the perception of and inference from analogy will ever vary amongst individuals according to their anterior knowledge and natural cast of thought. A series of facts will seem related to one set of ideas in the mind of one individual, and will present a comparative aspect totally different to another whose stock of information shall be differently arranged. It is direct relation alone, not that of resemblance among facts, that can establish any proposition bearing upon the economy of nature. Facts gathered from various sources, having certain general resemblances, do admirably well for the purpose of strengthening and elucidating any proposition, but when they are intended to serve for proof they should all present the same relation to the expected conclusion. So with phrenology. When the intention is to determine "scientifically" the function of any portion of the human cerebral mass, human heads should alone, in the first instance, supply the material for observation, and that which is noted invariably, under a given set of circumstances, may safely be treated as discovery. Then, if no error have taken place in conducting the observations, it will be certain to receive strength, and support, and elucidation from everything that natural history or comparative anatomy can afford. If it do not, the appeal to these sources of information will disprove the accuracy of the foregoing observations, and will lead to their rectification.

It is surprising to what erroneous conclusions a fault in method will lead even the most talented and the best informed. The mind under such circumstances, is with difficulty turned aside from the anticipated result, even on the presentation of the most glaring evidence. Who has forgotten the striking illustration afforded of this remark by Tiedemann who, some three or four years ago, took upon himself to prove that the negro race was not behind the European nations in general capacity or educability? From reflecting upon some vague analogies he had adopted the opinion that human perfectibility was in relation to the capacity of the cranial cavity. In order to determine accurately to what this capacity might amount he took a certain number of crania belonging to the different races and filled them with millet seed, and this being weighed furnished of course a true index of the relative size of each head. The entire assumption was wrong; it had been made without an adequate notation of relation between facts that were identical; and, having started badly, error accumulated itself upon error, and Tiedemann himself actually published a result that was at variance with his own figures. This was, at the time, well and clearly exhibited in the Phrenological Journal, by Dr. A. Combe, the substance of whose little memoir upon the subject was afterwards transferred to the pages of our own journal.

For hints respecting the various modes in which phrenology may be practically applied, other departments of human enquiry may very advantageously be consulted. The illustration of many of its facts and principles will be greatly aided by an appeal to other sources of information, and more particularly to general physiology and other allied branches of science. What has to be guarded against is the liability to mistake an elucidation of that which is already proved, for the just process of establishing some new conception; a proceeding frequently adopted, especially amongst merely literary phrenologists.

Highly as we estimate the discovery of Gall, immense as we regard the advantages which may be ultimately derived from phrenology, we confess that we wish to see it *less* regarded, studied, and pursued as a separate science, and *more* as a branch of general physiology. We are aware that the physiology of the brain is so inseparably identified with metaphysics as necessarily to constitute, under all circumstances, a special department; still, however, a certain distinctness might be maintained between the more strictly physiological and the metaphysical considerations of phrenology, which could but operate beneficially upon its future cultivation. A close attention to habits and pursuits, in association with specialities in the organization of brain, might go on, and the attempt to pursue these to their remote consequences be left to other hands. This latter is the business of the mental and moral philosopher. Gall might form the model of the former proceeding, whose method of dealing with the subject admits of positive verification or rectification by any one possessing the required accessory knowledge and appropriate opportunities; and a work like Mr. Combe's "Constitution of Man," might equally serve as an example for those who wished to illustrate, confirm or establish their particular views of human nature, by notions obtained from reflecting upon the physiology of the brain. Concerning the actual facts of phrenology, and its general axioms raised by the method of *induction*, there may ultimately, to a great extent, be concord and unanimity amongst its votaries. The *deductions* from these, on the part of individual phrenologists, will ever vary, according to their general views upon subjects in the discussion of which they apply its aids.

---

